# Early onset Colorectal Cancer and its association with its histological subtypes

**DOI:** 10.12669/pjms.40.10.10143

**Published:** 2024-11

**Authors:** Fatima Ibrar, Muslim Atiq, Farzana Shafqat, Hadi Mohammad Khan

**Affiliations:** 1 Fatima Ibrar, Departments of Gastroenterology Hepatology, Surgery and Pathology Shifa International Hospital, Islamabad, Pakistan; 2 Muslim Atiq, Departments of Gastroenterology Hepatology, Surgery and Pathology Shifa International Hospital, Islamabad, Pakistan; 3 Farzana Shafqat, Departments of Gastroenterology Hepatology, Surgery and Pathology Shifa International Hospital, Islamabad, Pakistan; 4 Hadi Mohammad Khan, Departments of Gastroenterology Hepatology, Surgery and Pathology Shifa International Hospital, Islamabad, Pakistan

**Keywords:** Colorectal cancer

## Abstract

**Objective:**

There are different clinicopathological features between early and late onset colorectal cancer. We aimed to characterize the histological features of colorectal cancer (CRC) at different age groups in our institution.

**Methods:**

Total 232 patients with histologically proven colorectal cancer between ages 13-99 were included. This is retrospective study. Data collected from tumour registry Shifa International Hospital from January 1^st^ 2018 to December 31^st^ 2020. Pearson Chi square test was used for significance of categorical variables. P-values less than 0.05 were considered significant.

**Results:**

Mean age at diagnosis was 55.49+/-16.43 years. Out of total 232 patients 58.6% were male and 41.3% were females (p value= 0.16). 150 (64.9%) were aged > 50 years and 81 (35.1%) were age < or equal to 50 years (p value = 0.44). Most common histological subtype was adenocarcinoma that was found in 188 (81%) cases. One hundred fifty five (66.8%) patients had Grade-II tumor, 67(28.9%) with Grade-III and 10 (4.3%) with Grade-I tumor. Fifty eight (25%) patients presented with metastatic disease. A significantly higher percentage of patients with signet ring cell cancer presented with a high-grade tumor when compared with patients with adenocarcinoma and mucinous carcinoma (93.7% vs 21.8%, p-value- <0.001%; 93.7% vs 39.2%, p-value <0.001). A significantly higher percentage of patients with mucinous adenocarcinoma and signet ring cell carcinoma presented at age less than 50 as compared to those with adenocarcinoma (60.7%, 56.2% and 30.8% respectively; p-value <0.05).

**Conclusion:**

This study signifies that mucinous and signet ring cell type CRC present at an early age and with a higher proportion of patients with high tumour grade. Early diagnosis is key to help improve outcomes in these patients.

## INTRODUCTION

Colorectal cancer (CRC) is one of the most feared manifestations of routine gastrointestinal complaints. The GLOBOCAN 2020 reported an estimated 19.3 million new cancer cases and 10 million cancer deaths in 2020 with lung cancer being the leading cause of death (18%) followed by CRC (9.4%) in males whereas in females, breast cancer was the most common cancer (11.7%) followed by lung (11.4%) and colorectal (10%) cancers respectively.^1^ Cancer has become a growing disease burden in Pakistan, posing a significant cause for concern. The World Health Organization has reported a rise in the incidence of cancer in Pakistan with incidence of colorectal cancer (4.9%).^2^

In Pakistan we do not have national tumor registry to determine the actual disease burden. However, Shaukat Khanum Memorial Hospital annual cancer registry report of 2021 showed that colon cancer is the second most frequently seen cancer after breast CA in Pakistan; and it constitutes for 7.7% of all malignancies.^3^ Patients with CRC require a thorough evaluation starting with clinical and biochemical assessments followed by radiological, endoscopic and histopathological assessments. Red flags for CRC include rectal bleeding, intractable diarrhea or new onset constipation and iron deficiency anemia. Approximately 49.3% patients had clinical features within three months of diagnosis.^4^ Development of CRC begins with benign adenomatous polyps and transforming through dysplasia, carcinoma in situ and then invasive carcinoma.^5^ Liver is the most common site of CRC metastasis due to hepatic portal venous system; and around 20% patients have distant metastasis at the time of presentation.^6^

In the recent years, it has been observed that CRC incidence is increasing in developing countries due to change in lifestyle and dietary habits.^7^ Pakistan has been historically considered a low-risk country regarding colorectal cancer but changes in epidemiology warrants us to implement screening so that colorectal cancer can be diagnosed at an earlier stage with outcome.^8^ Colorectal cancer is one of the most common cause of cancer related deaths and most of patients die in first five years after diagnosis.^9^ Therefore, early diagnosis is key to improving outcomes in CRC. In our set up, where there seems to be a considerable lag between when symptoms first appear and when the related diagnostic evaluation begins, it is perhaps even more important to encourage endoscopic evaluation in a timely fashion, especially in the younger age group.

Early onset CRC group has been seen to be associated with a higher incidence of poorly differentiated cases, with advanced stage, mucinous or signet ring cell type.^10^ The data on histological subtypes of CRC in our setting is rather scarce. This study was designed to further investigate the association of disease onset and disease activity in CRC patients in our setting especially with reference to the histological subtypes of CRC.

## METHODS

This retrospective study was conducted at Shifa International Hospital (SIH), Islamabad. Data on all patients belonging to Pakistan who were evaluated for CRC at SIH between January 2019 and December 2021 was extracted from the tumor registry. Cases that were misclassified in the database as CRC were excluded. Cases with missing information of tumor grading or staging were also excluded. Fifty years of age was taken as cut off to be “young as conventional literature on CRC recommended screening starting at age 50 years. Variables in study were age, location of tumour grade and stage of tumour and histology type. Grade I/II histology was reclassified as low grade whereas Grade-III was classified as high grade histology.

### Ethical Approval:

It was obtained from the Institutional Review Board number 0195-22; dated August 11, 2022.

### Statistical Analysis:

Data was analysed using SPSS version 29.0. Difference in mean was determined using independent sample t-test. Chi square test was used for significance of categorical variables. P-values less than 0.05 were considered significant.

## RESULTS

**Fig.1:**
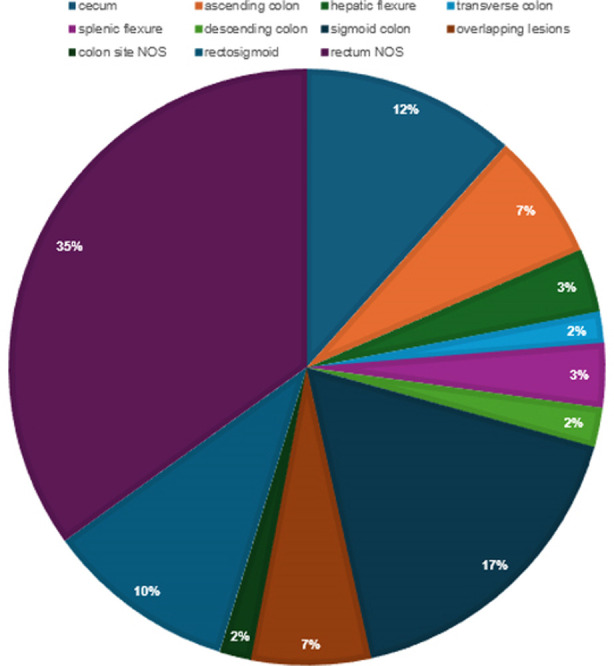
Distribution of CRC.

Total of number of cases extracted from the tumor registry were 232. Mean age was 55 ± 16.4 years. There were 136 males (58.6%) and 96 females (41.3%). Most common histological variant was adenocarcinoma, followed by mucinous carcinoma and signet ring cell carcinoma respectively. One hundred fifty five(66.8%) had Grade-II tumor on histology grading scale, followed by 67(28.9%) with Grade-III and 10(4.3%) with Grade-I. Fifty eight (25%) patients presented with metastatic disease. (Table-I) Most of the tumors were located on the left side of the colon (Fig.1). Patients with mucinous carcinoma were younger than patients with adenocarcinoma (49.11± 16.4 years vs 57.39 ± 15.92 years; p-value 0.01). Patients with signet ring carcinoma were younger than patients with adenocarcinoma (44.38± 16.19 years vs 57.39± 15.90 years; p-value 0.02). There was no significant difference in age between patients with mucinous carcinoma and signet ring cell carcinoma (49.11 ± 16.40 years vs 44.38± 16.19 years; p-value 0.77).

**Table-I:** Baseline characteristics of patients.

***Age***	
Mean +/- SD	55.5 +/- 16.4 years
<50 years	84 (36.2%)
>50 years	148 (63.8%)
***Sex***	
Male	136 (58.6%)
Female	96 (41.3%)
***Histological Subtypes***	
Adenocarcinoma	188 (81%)
Mucinous Carcinoma	28 (12.1%)
Signet Ring Cell Carcinoma	16 (6.9%)
***Histological Grades***	
G1	10 (4.3%)
G2	155 (66.8%)
G3	67 (28.9%)
***AJCC Staging***	
Stage I	11 (4.7%)
Stage II	54 (23.3%)
Stage-III	109 (47.0%)
Stage IV	58 (25.0%)

**Fig.2:**
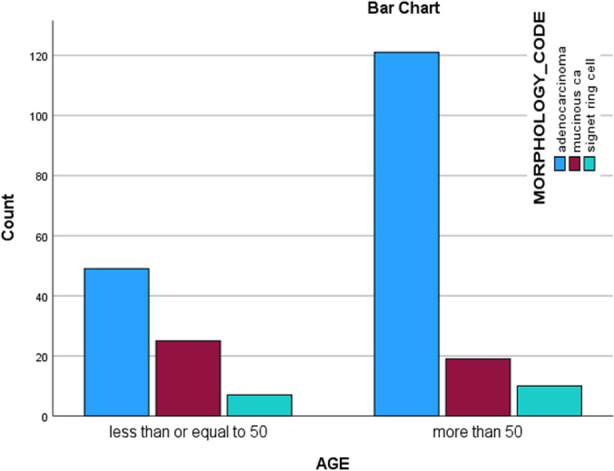
Comparison of early onset vs late onset CRC with regards to histology.

A significantly higher proportion of patients with mucinous carcinoma and signet ring cell carcinoma presented at age <50 years as compared to patients with adenocarcinoma (p-value <0.05) (Table-II). A significantly higher proportion of patients with signet ring cell carcinoma presented as G3 or poorly differentiated tumors as compared to those with adenocarcinoma and mucinous carcinoma (93 % vs 22 %, p-value; <0.001% vs 59 %, p-value <0.001).

**Table-II:** Age and histological grade comparison between CRC histological subtypes.

Histology Subtypes	N	Age in years (mean)	SD (+/-)
Adenocarcinoma	188	57.3	15.9
Signet Ring Cell Carcinoma	16	44.4	16.1
Mucinous Carcinoma	28	49.1	16.4

Total	232	55.5	16.434

## DISCUSSION

In our study, more than one third of patients with adenocarcinoma (69%) were of age > 50 years and less than one-third (31%) were of age < 50 years (Fig.1). More than three fourth of these patients (78%) had low or intermediate grade tumour at presentation. As reported in the literature, the most common histology in our patient with CRC was adenocarcinoma.^11^ Most colorectal cancers are classified as adenocarcinomas, consisting of the “garden variety” adenocarcinoma, and its variants including the mucinous and signet ring adenocarcinoma. It is typically characterized by cribriform glands lined with atypical cells.

Second common histological variant in our study was mucinous carcinoma. It is characterized by large mucin lakes in tumour area seen on histology. In our study, around 60% of these patients were found to have a low or intermediate grade tumour and the same proportion (60%) presented at age < 50 years. Mucinous histology is a negative prognostic factor in young patients with colorectal cancer. This is associated with early and high recurrence rates, despite use of standard neoadjuvant and adjuvant regimens.^12^

Signet ring cell carcinoma is very rare ranging from <1 to 2.4%.^13^ In contrast, signet ring cell type carcinoma constituted 6.8% of all adenocarcinomas. It is characterized by signet ring shaped cells with intra cellular mucin displacing nucleus to one side. About 60% these patients were of age < 50 years; and almost all (93.7%) of these cases were poorly differentiated. It is comparable with global data where signet ring cell cancer is seen in younger population with high grade at presentation; and is associated with poor prognosis.^14^ This underscores the importance of further diagnostic evaluation for lower gastrointestinal symptoms in our population, especially in younger patients so these lesions could potentially be detected at an early stage.

Majority of our study population presented with stage-III (46.9%) disease, suggesting at least locally advanced stage in majority of our CRC patients at the time of presentation. This could be reflective of a general lack of awareness about symptoms depicting CRC; and the availability of screening programs for CRC in our population. These results are comparable to global data where symptomatic patients were also noted to have advanced stage at presentation.^15^

In our study, the mean age of patients with colorectal cancer was 55 years in contrary to a recent study conducted in Pakistan where mean age was less than 50 years.^16^ Those authors also noted that the mean age at diagnosis for right side colon cancer patients was 43.9 years and for left side colon cancer, it was 49.8 years. In their observation, right sided cancer was more likely to be poorly differentiated.^16^ Similar observation about earlier diagnosis of CRC in Pakistan have been made before.^17^,^18^ In recent years worldwide incidence of CRC is increased in younger population with age < 50 years.^19^ Along the same lines, when right sided CRC is compared with left side CRC, right sided CRC is noted to have increased metastatic potential.^20^

It has been reported that the disease-free survival and overall survival are significantly lower in the mucinous adenocarcinoma group than in the non-mucinous adenocarcinoma group. In subgroup analysis, this difference is only noted in stage-III colon cancer.^21^

It has also been shown that in patients with a resectable, nonhereditary, primary colorectal signet ring cell carcinoma, the disease-free survival and the overall survival rates were significantly lower in the signet ring group compared to the adenocarcinoma group. In a subgroup analysis stratified by stage however, signet ring histologic type could be regarded as an independent poor prognostic factor when the cancer was in an advanced stage, with worse overall survival and disease-free survival. This was quite in contrast to an early stage of diagnosis when no such difference was observed between the two groups.^22^ Therefore, it has been well established that survival outcomes are comparable for histological subtypes of CRC when diagnosed at early stages (stage-II or less).^23^

These findings become quite consequential in our setting when most patients present at stage-III or later. Therefore, our study also highlights the importance of an early diagnosis of CRC based on high index of suspicion so that cases can be diagnosed and treated promptly at an early stage. This is especially important in those patients presenting with red flags such as rectal bleeding or weight altered bowel habits with weight loss, at a young age. Therefore, we must shift our focus on improving awareness about the alarm symptoms of CRC so that patients themselves take these symptoms seriously and report to health care professionals in a timely manner. Delay in diagnosis leads to the more advanced disease, hence poor prognosis.

### Strengths:

To our knowledge this is first study of Pakistan that corroborates the age at the time of diagnosis of CRC with its histological subtypes. This data is unique in the sense that it relates histological subtypes of CRC with tumour biology as well as age at presentation.

### Limitations

Our main study limitation was that this analysis was based on retrospective data that was acquired from a single institution.

## CONCLUSION

This study signifies that mucinous and signet ring cell type CRC present at an early age and with a higher proportion of patients with high tumour grade. Early diagnosis is key to help improve outcomes in these patients.

### Authors’ Contribution:

**FI:** Substantial contribution to study design, data acquisition, manuscript writing and revising it critically.

**MA:** Conception, study design, review and approval of final version.

**FS:** Data analysis and Interpretation.

**HMK:** Manuscript writing

## References

[1] Sung H, Ferlay J, Siegel RL, Laversanne M, Soerjomataram I, Jemal A (2021). Global Cancer Statistics 2020:GLOBOCAN Estimates of Incidence and Mortality Worldwide for 36 Cancers in 185 Countries. CA Cancer J Clin.

[2] Tufail M, Wu C (2023). Exploring the Burden of Cancer in Pakistan:An Analysis of 2019 Data. J Epidemiol Glob Health.

[3] Annual Cancer Registry Report 2022 Retrieved from https://shaukatkhanum.org.pk/wp-content/uploads/2023/05/Annual-Cancer-Registry-Report-2022pdf.

[4] Mahmoud NN (2022). Colorectal Cancer:Preoperative Evaluation and Staging. Surg Oncol Clin N Am.

[5] Wu Z, Li Y, Zhang Y, Hu H, Wu T, Liu S (2020). Colorectal Cancer Screening Methods and Molecular Markers for Early Detection. Technol Cancer Res Treat.

[6] Zhao W, Dai S, Yue L, Xu F, Gu J, Dai X (2022). Emerging mechanisms progress of colorectal cancer liver metastasis. Front Endocrinol (Lausanne).

[7] Abro MN, Khan ME, Ibrahim M, Maheshwari GK, Asghar G, Ahmed S (2022). Incidence of Colorectal Carcinoma in the Remote Area of Sindh, Pakistan. Adv Life Sci.

[8] Tayyab GN, Dilshad A, Toor I, Rasool S, Hassan GU, Jabbar S (2023). National Screening Program for Colorectal Cancer. Pak Postgrad Med J.

[9] Ali I, Fayyaz A, Anwar Z, Saif I, Khan I, Jahangir M (2021). Mutation analysis of TP53 in colorectal cancer, Peshawar, Khyber Pakhtunkhwa, Pakistan. J Clin Med Kaz.

[10] Gao XH, Li J, Liu LJ, Zheng NX, Zheng K, Mei Z (2022). Trends, clinicopathological features, surgical treatment patterns and prognoses of early-onset versus late-onset colorectal cancer:A retrospective cohort study on 34067 patients managed from 2000 to 2021 in a Chinese tertiary center. Int J Surg.

[11] Kakar S, Smyrk TC (2005). Signet ring cell carcinoma of the colorectum:Correlations between microsatellite instability, clinicopathologic features and survival. Mod Pathol.

[12] Soliman BG, Karagkounis G, Church JM, Plesec T, Kalady MF (2018). Mucinous Histology Signifies Poor Oncologic Outcome in Young Patients with Colorectal Cancer. Dis Colon Rectum.

[13] Pozos-Ochoa LI, Lino-Silva LS, Leon-Takahashi AM, Salcedo-Hernandez RA (2018). Prognosis of Signet Ring Cell Carcinoma of the Colon and Rectum and their Distinction of Mucinous Adenocarcinoma with Signet Ring Cells. A Comparative Study. Patholo Oncol Res.

[14] An Y, Zhou J, Lin G, Wu H, Cong L, Li Y (2021). Clinicopathological and Molecular Characteristics of Colorectal Signet Ring Cell Carcinoma:A Review. Pathol Oncol Res.

[15] Moreno CC, Mittal PK, Sullivan PS, Rutherford R, Staley CA, Cardona K (2016). Colorectal Cancer Initial Diagnosis:Screening Colonoscopy, Diagnostic Colonoscopy, or Emergent Surgery, and Tumor Stage and Size at Initial Presentation. Clin Colorectal Cancer.

[16] Khan SZ, Fatima I (2019). Tumor sidedness and clinicopathological features of resected colon cancer in rural population of Northern Pakistan:single institution analysis. J Colpoproctol.

[17] Anwar N, Badar F, Yusuf MA (2008). Profile of patients with colorectal cancer at a tertiary care cancer hospital in Pakistan. Ann New York Acad Sci.

[18] Zahir MN, Azhar EM, Rafiq S, Ghias K, Shabbir-Moosajee M (2014). Clinical features and outcome of sporadic colorectal carcinoma in young patients:a cross-sectional analysis from a developing country. Int Sch Res Notices. 2014.

[19] Spaander MCW, Zauber AG, Syngal S, Blaser MJ, Sung JJ, You YN (2023). Young-onset colorectal cancer. Nat Rev Dis Primers.

[20] Zheng C, Jiang F, Lin H, Li S (2019). Clinical characteristics and prognosis of different primary tumor location in colorectal cancer:a population-based cohort study. Clin Transl Oncol.

[21] Kim S, Huh JW, Lee WY, Yun SH, Kim HC, Cho YB (2023). Prognostic Impact of Mucinous Adenocarcinoma in Stage II and III Colon Cancer. Dis Colon Rectum.

[22] Yun SO, Cho YB, Lee WY, Kim HC, Yun SH, Park YA (2017). Clinical Significance of Signet-Ring-Cell Colorectal Cancer as a Prognostic Factor. Ann Coloproctol.

[23] Wu X, Lin H, Li S (2019). Prognoses of different pathological subtypes of colorectal cancer at different stages:A population-based retrospective cohort study. BMC Gastroenterol.

